# BrDMrisc – Online Brazilian Risk Score for Incident Type 2 Diabetes – ELSA-Brasil Cohort

**DOI:** 10.11606/s1518-8787.2025059007088

**Published:** 2026-01-09

**Authors:** Paula Andreghetto Bracco, Maria Inês Schmidt, Alvaro Vigo, José Geraldo Mill, Pedro Guatimosim Vidigal, Sandhi Maria Barreto, Mária de Fátima Sander Diniz, Maria de Jesus Mendes da Fonseca, Bruce Bartholow Duncan

**Affiliations:** I Universidade Federal do Rio Grande do Sul. Faculdade de Medicina. Programa de Pós-Graduação em Epidemiologia. Porto Alegre, RS, Brasil; II Universidade Federal do Rio Grande do Sul. Instituto de Matemática e Estatística. Departamento de Estatística. Porto Alegre, RS, Brasil; III Universidade Federal do Rio Grande do Sul. Hospital de Clínicas de Porto Alegre. Porto Alegre, RS, Brasil; IV Universidade Federal do Espírito Santo. Centro de Ciências da Saúde. Vitória, ES, Brasil; V Universidade Federal de Minas Gerais. Faculdade de Medicina. Belo Horizonte, MG, Brasil; VI Universidade Federal de Minas Gerais. Hospitais das Clínicas. Belo Horizonte, MG, Brasil; VII Fundação Oswaldo Cruz. Escola Nacional de Saúde Pública. Rio de Janeiro, RJ, Brasil

**Keywords:** Diabetes Mellitus, Type II, Diagnostic Screening Program, Brazil, Incidences

## Abstract

**OBJECTIVE:**

To describe the Brazilian Diabetes Risk Score (BrDMrisc), an online calculator for estimating the risk of developing diabetes in 10 years, comparing it to other commonly used screening approaches to identify high-risk of future diabetes.

**METHODS:**

BrDMrisc is derived from 7.4-year follow-up data from ELSA-Brasil, a contemporary Brazilian cohort. Risk functions to predict future diabetes were based on socioeconomic, lifestyle, clinical, and laboratory variables, many of which are incorporated as continuous variables on a training sample including half of the analyzed cohort. We used ELSA-Brasil participant baseline data and incident diabetes cases detected during follow-up on the other half to compare the predictive capacity of BrDMrisc against traditional pre-diabetes screening strategies.

**RESULTS:**

BrDMrisc’s 27 risk functions offer versatility to the online app, enabling estimates based on differing combinations of clinical and laboratory findings, which present more favorable diagnostic properties than other recommended approaches. For example, the BrDMrisc function based on clinical data plus fasting glucose, when compared with detection using solely the presence of impaired fasting glucose (≥ 100 mg/dL), identified a more manageable fraction (20.0% *versus* 40.6%) of the population as high risk, with those identified as presenting a two-fold risk of developing diabetes (28.5% *versus* 17.1%).

**CONCLUSIONS:**

BrDMrisc diabetes risk calculator, based on data from a contemporary Brazilian cohort, is readily available online, versatile, and presents generally more favorable diagnostic properties than other commonly used screening strategies.

## INTRODUCTION

Brazil, like most low and middle-income countries, has witnessed a marked rise in the burden of diabetes and hyperglycemia over recent decades. The International Diabetes Federation estimated a 10.5% prevalence of diabetes in Brazilian adults in 2021, with 31.9% of the cases being undiagnosed^1^. The Global Burden of Disease study estimated from 2036 onward, type 2 diabetes will be Brazil’s leading cause of disease burden^2^.

Clinical trials have shown lifestyle interventions and medications to be effective when administered to individuals with an abnormal oral glucose tolerance test (OGTT)^3,4^. While cost-effectiveness analyses suggest that improvements in screening and treatment are necessary to support population-wide screening and diabetes prevention programs^5,6^, it is undeniable that preventing diabetes in high-risk individuals can reduce its burden. On this basis, primary prevention performed by identifying high-risk individuals for preventive actions to decrease disease burden is frequently recommended as a significant preventive strategy. However, when evaluated, the populational reach of these interventions has been low^7,8^. One key reason for this scenario is the difficulty in identifying high-risk individuals as targets for diabetes prevention. To facilitate this task, risk scores have been developed to predict for whom glycemic testing is indicated and, with testing, those at sufficiently high risk of developing diabetes to merit intervention^9^. Although simple scores based on easily obtainable clinical data are recommended^10,11^, low-cost and easy first step to identifying high-risk individuals in the population, such as using laboratory glycemia measures, especially when combined with these clinical factors, has considerably higher predictive capacity^12^.

Few scores for detecting diabetes risk have been developed in Latin America^13^, with only two in Brazil. Derived from a sample of 1,224 adults living in Vitória, Espírito Santo, the first Brazilian score contained only clinical variables and was created based on its ability to detect prevalent diabetes as ascertained by fasting glucose^14^. To detect those at high risk of developing diabetes, the second score was derived from the *Estudo Longitudinal da Saúde do Adulto* (ELSA-Brasil - Brazilian Longitudinal Study of Adult Health) cohort^15^. Based on this cohort of 9,525 adults (aged 35 to 74), we showed that a score based on continuous clinical and laboratory values presented diagnostic properties generally superior to those of the recommended screening approaches^16^.

In this report, we compared the diagnostic properties and applicability of this ELSA-Brasil score, here named the Brazilian Diabetes Risk Score (BrDMrisc), with two other commonly recommended screening approaches in Brazil: a fasting plasma glucose (FPG) ≥ 100 mg/dL and a two-step approach based on the Finnish Diabetes Risk Score (FINDRISC) questionnaire, showing why BrDMrisc outperforms the others. We also briefly illustrate its applicability using the freely available BrDMrisc calculator in two screening moments.

## METHODS

### Development of the BrDMrisc Algorithms and App

ELSA-Brasil is a multicenter cohort study that enrolled 15,105 employees from universities and research institutions in six different Brazilian states, from 2008 to 2010^10,12^. Subsequent visits occurred in 2012–2014 and 2016–2018^17^, producing an average 7.4-year follow-up. The Research Ethics Committee of each investigation center approved ELSA-Brasil, and all participants gave written consent to participate.

Demographic, socio-economic, lifestyle, and anthropometric data were obtained using standardized questionnaires and protocols. Diabetes was ascertained by self-report, use of antidiabetic drugs, or by at least one of three laboratory measures: fasting plasma glucose ≥ 126 mg/dL (7 mmol/L), 2h glucose ≥ 200 mg/dL (11.1 mmol/L) by standard oral glucose tolerance test, or glycated hemoglobin (HbA1c) ≥ 6.5% (48 mmol/mol). Cases were considered prevalent at baseline if positive for any of these criteria. Verification of incident cases required at least one of these five criteria at both follow-up visits or at least two at a single visit. We excluded participants with no diabetes criteria present at the first follow-up and only one of the criteria at the second follow-up, as we had no means of detecting their later confirmation. We did not include as cases of incident diabetes those who met two criteria at the first follow-up but none at the second follow-up.

To initiate analyses, we randomly divided our sample in half, creating training and validation datasets. We applied logistic regression to the training dataset to develop risk functions for predicting incident diabetes. We initially produced models with only socio-demographic and non-laboratory clinical data (age, sex, ethnicity, parental history of diabetes, hypertension, body mass index, and waist circumference; “clinical model”) and then added laboratory measurements. We identified variables to remain in models that significantly improved detection, as evaluated by the area under the receiver-operating characteristic curve. We investigated possible non-linear effects for all of our continuous variables by testing the addition of their quadratic term in the models, using the Wald test and considering a significance level of 5%. Next, we applied the logistic regression coefficients from the various derived equations to the validation dataset to estimate each participant’s probability of developing diabetes. The development and validation of the predictive equations have previously been described in greater detail^16^.

We graphically showed and compared the diagnostic properties of the different testing options available with the BrDMrisc online calculator, considering as high risk, for each equation, those with a probability of developing diabetes within 10 years ≥ 20%. Four key diagnostic properties were estimated: the percentage of sample labeled as high risk, the percentage of cases that screening detects (sensitivity), and the probability that an individual, once labeled as high risk or not, will actually develop diabetes (positive and negative post-test probability, respectively).

Next, we compared these diagnostic properties of the BrDMrisc approach, when based on clinical variables and fasting glucose (FPG), with the pre-defined cut-points of established strategies: the American Diabetes Association (ADA) definition of impaired fasting glucose of ≥ 100mg/dL and the finnish approach of an oral glucose tolerance test fasting glucose ≥ 110mg or 2h glucose of ≥ 140mg/dL following a positive FINDRISC questionnaire.

### Ethics and Consent to Participate

The ELSA-Brasil study was approved by the *Comitê de Ética em Pesquisa do Hospital de Clínicas de Porto Alegre*. All participants provided their written informed consent to participate in this study.

### Online Tool

Based on the equations developed, we built an online App using R Shiny to calculate the 10-year risk of developing diabetes. To transform probabilities of incident diabetes based on our 7.4-year average follow-up (7yrProb) into 10-year risk (10yrProb), we applied the following equation^18^:


$$10yr\mathrm{Prob}=1-\left[\exp\left(-\left(-\frac{\log(1-7yr\text{ Prob })}{7.4}\right)\times 10\right)\right]$$

The app is freely available on the ELSA-Brasil website^a^.

## RESULTS

We excluded 2,429 participants with diabetes at baseline, 25 due to missing information to confirm diabetes or for missing the variables considered to build the risk score, 1,971 due to loss to follow-up, and 1,155 with missing or uncertain outcomes (i.e., with only one of the criteria for diabetes for cases detected at the second follow-up). Following exclusions, the sample used to construct the BrDMrisc equations included 9,525 participants randomly divided into training and validation samples. Training and validation datasets had similar distributions of variables considered in building the risk score and a similar (9.1%) incidence of diabetes over the 7.4-year follow-up^16^.

**Figure 1:**
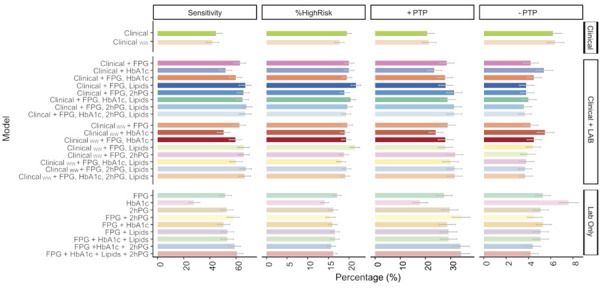
Diagnostic characteristics of the 27 risk models comprising BrDMrisc as evaluated in the validation dataset.

Note: %HighRisk: probability of developing diabetes over 10 years ≥ 20%; Sensitivity: percent of participants with future diabetes identified as high risk; +PTP (positive post-test probability): probability of developing diabetes among those labeled as high-risk; −PTP (negative post-test probability): probability of developing diabetes among those not labeled; ww: models without waist circumference information; Lab: laboratory; FPG: fasting plasma glucose; 2hPG: 2 hours plasma glucose.

Figure 1 describe the sensitivity, the percent of the sample labeled as high-risk, the probability of those labeled as high-risk to develop diabetes (positive post-test probability; +PTP), and the probability of those not at high risk to develop diabetes (negative post-test probability; -PTP) for clinical, laboratory, and combined data enabling calculation using the BrDMrisc functions. Comparing these properties allows us to evaluate the pros and cons of the different strategies. We observed that removing waist circumference from the clinical data (Clinical without waist or Clinical ww) decreases sensitivity while increasing the percent labeled, albeit slightly. Additionally, combining glucose (fasting or 2hPG) values with clinical information produces more favorable diagnostic properties. FPG alone has notably lower sensitivity. Compared with clinical data alone, adding FPG improves the sensitivity and +PTP while lowering -PTP without much change in the percentage identified as high risk. Adding additional laboratory findings to this combination enhances the predictive values of the scores.

**Figure 2:**
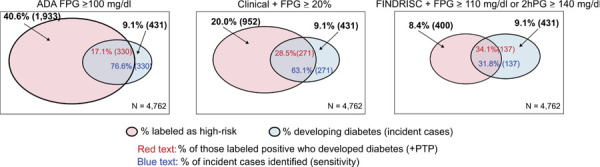
Comparison of three diagnostic strategies.

Note: Center: BrDMrisc with positivity defined as those with a probability of developing diabetes over 10 years ≥ 20%; Left: the American Diabetes Association (ADA) strategy of testing all aged ≥ 35 years with a fasting glucose (FPG); and Right: the Finnish strategy of first using the FINDRISC questionnaire and then, for all found positive, applying an oral glucose tolerance test with fasting and 2h glucose (2hPG). In each graph, the blue circle represents the 9.1% who developed diabetes during follow-up. The red number is the percentage of the ELSA-Brasil sample labeled as at high risk (the pink circle) who developed diabetes (the post-test probability), and the blue number is the percentage of the incident cases that the strategy identified (sensitivity).

Figure 2 graphically describes these BrDMrisc diagnostic properties when based on clinical variables and FPG, comparing them to those of two other strategies recommended in Brazil. First is an FPG ≥ 100mg/dl, the simplest of screening options recommended by the ADA and the Brazilian Society for Diabetes^19,20^ for all adults aged 35 or more. Here, high risk (pre-diabetes) is considered positive if impaired fasting glucose (FPG ≥ 100mg/dL) is detected, independent of clinical data. The second is the two-step strategy based on the FINDRISC questionnaire. Here, the approach is first to screen with the FINDRISC questionnaire and, if positive, then test with an OGTT, with an FPG ≥ 110mg/dL (impaired fasting glucose as defined by the World Health Organization [WHO]) or 2h glucose ≥ 140mg/dL (impaired glucose tolerance) indicating high risk^11,21^.

In these area graphs, the pink circles represent the percentage of the ELSA sample labeled as high risk by the three different screening strategies, and the blue circles represent the 9.1% who developed diabetes during follow-up. Within the overlapping parts of the circles, the number in red is the probability of a labeled individual developing diabetes (positive post-test probability, +PTP), and the number in blue is the percentage of future cases identified by the screening strategy (sensitivity). Important differences can be seen in the fraction of the sample labeled high-risk, from a low 8.4% for the FINDRISC strategy to a much higher 40.6% for the presence of ADA-defined impaired fasting glucose, with the BrDMrisc approach resulting in an intermediate proportion of 20% (the fact that the BrDMrisc cutoff for high risk and the sample fraction labeled as high-risk are both 20% is merely a chance occurrence). ADA-defined impaired fasting glucose identified the greatest fraction of future cases (76.6%), a little over the 63.1% estimated by BrDMrisc and considerably more than the 31.8% detected with the FINDRISC approach. The risk of developing diabetes among those labeled as high-risk was considerably lower with the ADA approach (17.1%) than with FINDRISC (34.1%) or BrDMrisc (28.5%).

Table 1, which presents the predicted risk resulting from differing body mass index (BMI) and fasting glucose levels, shows why it is logical that BrDMrisc, which expresses these and other variables continuously in calculations, can identify a large fraction of cases (different from the FINDRISC strategy) without an excessively high fraction being labeled as high risk (different from the ADA strategy). The 10-year risk of developing diabetes for hypothetical cases with otherwise fixed clinical characteristics (as described in the table footnotes) went from 10% when BMI was 23kg/m^2^ to 30% at 40kg/m^2^. Similarly, the 10-year risk of developing diabetes for hypothetical clinical cases with otherwise fixed values of clinical risk factors ranged 10-fold, from 6% when fasting glucose was 95mg/dl to 63% at 123mg/dL. When FPG was analyzed categorically, the 10-year risk for the described clinical case ranged less than 2-fold, from 21.6% if prediabetes was present (FPG ≥ 100mg/dL) to 12.1% if absent (FPG < 100mg/dL).

**Table 1:** Variability in the ten-year risk of developing diabetes (post-test probability) for hypothetical patients as a function of differing body mass index (BMI) and fasting glucose values. ELSA-Brasil.

Characteristic	Clinically relevant range												
BMI (kg/m^2^)	23	25	27	28	29	30	31	32	33	34	35	37	40
10-year risk (%)^a^	10	12	13	14	15	16	18	19	20	21	23	26	30
FPG (mg/dL)	95	98	100	102	104	106	108	110	112	114	116	120	123
10-year risk (%)^b^	6	8	10	12	15	18	22	26	31	36	42	54	63

^a^ For a White man aged 50 with a waist circumference of 100cm, without hypertension or a family history of diabetes with the different BMI values as indicated in the row, the score being based just on these clinical variables.

^b^ For a White man aged 50 with a BMI of 28 kg/m^2^ and a waist circumference of 100cm, without hypertension or a family history of diabetes, with the different fasting glucose values as indicated in the row.

Note: standard cutoffs are indicated in bold.

Similarly, Table 2 summarizes the 10-year risk predicted by the same fasting glucose value in hypothetical cases presenting a wide range of clinical risk, whether using the ADA cutoff point (100mg/dL) or the WHO (110mg/dL) to define high risk. Depending on clinical characteristics, the risk can vary from 6% to 20% for someone with an FPG = 100mg/dL and from 16% to 46% for someone with an FPG = 110mg/dL. However, based solely on a categorical ADA or WHO classification of fasting glucose, all of these cases would be considered pre-diabetes and thus high-risk.

**Table 2:** Variability in the ten-year risk of developing diabetes (post-test probability) for hypothetical patients with impaired fasting glucose (FPG = 100 mg/dL or 110mg/dL) but with varying risks (pre-test probabilities) based on the clinical characteristics of their presentation.

Clinical Characteristics													
Age (years)	35	35	40	40	40	40	45	40	45	45	50	53	55
BMI (kg/m^2^)	23	23	27	29	28	30	31	31	32	32	32	32	33
Waist (cm)	88	98	98	98	98	98	99	100	100	103	104	104	105
Hypertension	-	-	-	-	-	-	-	+	+	+	+	+	+
Family history	-	-	-	-	+	+	+	+	+	+	+	+	+
**Probability (%) of developing diabetes in 10 years**													
Pre-test													
Just clinical	5	7	10	12	15	17	20	22	25	27	30	32	35
Post-test													
+FPG = 100mg/dL^a^	6	7	9	10	12	14	16	16	18	18	19	19	20
+FPG = 110mg/dL^b^	16	18	24	27	31	35	37	39	41	42	43	43	46

BMI: body mass index; Waist: waist circumference; Family history: family history of diabetes in mother, father, or sibling; FPG: fasting glucose.

^a^ Cutoff recommended by the American Diabetes Association.

^b^ Cutoff recommended by the World Health Organization.

Note: estimated 10-year risk of developing diabetes of a 44-year-old white woman with hypertension but without a family history of diabetes, having a body mass index of 28kg/m^2^ and a waist circumference of 88cm. Her risk was estimated initially (Panel A) based only on these clinical variables, and then finally (Panel B) after a subsequent fasting glucose of 113mg/dL was obtained.

**Figure 3:**
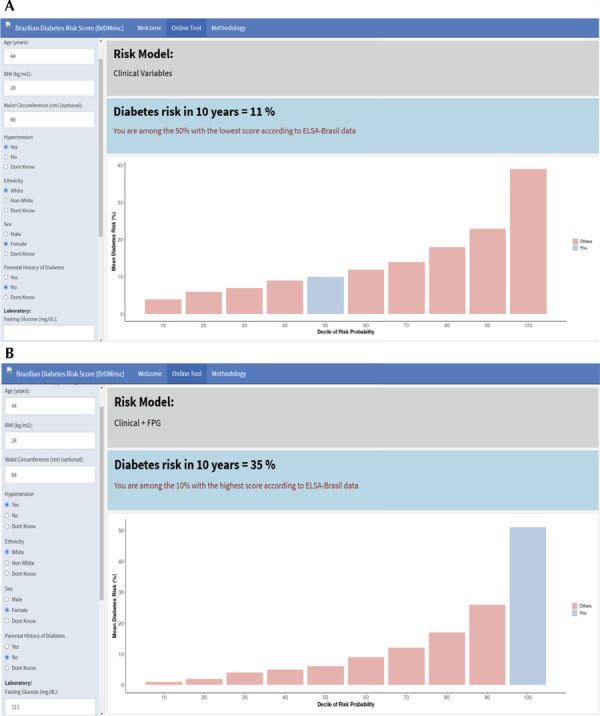
Input and output of the online BrDMrisc calculator showing the estimated 10-year risk of developing diabetes.

Risk estimates from the BrDMrisc can be readily obtained by accessing its online calculator. Figure 3 presents two such examples, using as a clinical case a hypothetical 44-year-old White woman with a BMI of 28kg/m^2^, waist circumference of 88cm, with self-reported hypertension but without a family history of diabetes. Entering these values and clicking “run,” her 10-year risk of developing diabetes (Panel A) was estimated as 11%, probably too low to justify more than general health counseling but high enough to justify proceeding to laboratory testing. If upon testing she presented a fasting glucose of 113mg/dL, her risk would rise to 35% (Panel B), a level justifying preventive treatment.

## DISCUSSION

The BrDMrisc calculator presents several favorable characteristics. Risk can be calculated based on clinical characteristics, laboratory results, or both. It maximizes diagnostic accuracy by using the continuous values of the available clinical and laboratory measurements in its calculations. Its output, the probability of developing diabetes in 10 years, is a more transparent and informative result than just the presence of impaired fasting glucose. Finally, being accessible via the internet, it is readily available for quick calculations, whether by individuals wishing to know their risk or by clinicians or clinical staff identifying patients for intervention.

While scores are generally derived from associations that vary little across geographies, they can require calibration to the disease incidence (here diabetes) to provide accurate results for a population distinct from that in which the score was developed. As this score was derived from ELSA-Brasil, a cohort containing multiple domains of Brazilian society in terms of age, sex, ethnicity, and schooling level, it should serve the Brazilian population well. While the FINDRISC questionnaire has been carefully translated and transculturally adapted^22^, independent external validation of the original FINDRISC model has been performed in Latin American countries and most studies used the same predictors to fit new predictive models^23^. Given the lack of screening tests that have been fully developed and validated in Latin American countries^13^, BrDMrisc may be better calibrated to most low- and middle-income countries than commonly recommended scores.

An essential feature of BrDMrisc is that its output is a defined 10-year probability of developing diabetes. This, as noted, differs from scores that define positive as impaired fasting glucose for which, as shown here, the likelihood of developing diabetes can vary considerably as a function of the underlying risk conferred by the clinical characteristics. The 20% cutoff to define high risk means that those who screen positive will have a 20% chance of developing diabetes within 10 years. In contrast, the risk of developing diabetes based on just an FPG ≥ 100mg/dL is not apparent.

Our choice of a 20% risk of developing diabetes as the cutoff for presenting its diagnostic properties was somewhat arbitrary, as it was not defined by any authority. Adopting this cutoff point clinically would mean that those above 20% risk should receive preventive interventions. In the ELSA sample, this cut-off point is higher than the 10-year risk of a fasting glucose of 100mg/dL (10%), slightly higher than that of an OGTT 2h glucose of 140mg/dL (17.6%), and close to the 21% we found in our sample for the NICE two-step strategies^16^. However, 20% is somewhat lower than the cumulative incidence of all those in the ELSA sample with impaired glucose tolerance (2h glucose ≥ 140 mg/dL; 26.2%), the entrance criteria for the two classic diabetes prevention clinical trials studies^24,25^ that justified screening by showing a 58% reduction in diabetes incidence with post-screening lifestyle interventions. In any case, the BrDMrisc, by outputting the probability of developing diabetes, leaves the cut-off for screen positivity that merits intervention for the user to define.

Thus, while defining screen positivity by a fasting glucose cut-off of 100mg/dl, regardless of clinical variables, identified more cases (76.6%) than the BrDMrisc approach (63.1%), this isolated impaired fasting glucose strategy identified in 40.6% of the sample as high-risk against the 20% identified by the BrDMrisc approach. Additionally, the ADA high-risk group consisted mainly (83%) of individuals who would not progress to diabetes over the ensuing 10 years. Systematically providing intervention for all of them would require a large resource allocation, in addition to the individual burden. In other words, using continuous, as opposed to categorical variables, the BrDMrisc produced a more accurate estimate than scores based on categorical variables, facilitating the allocation of clinical resources for eventual interventions.

The versatility of the online BrDMrisc calculator derives from its calculations based on 27 different combinations of variables, ranging from just clinical or laboratory ones to those combining clinical variables with laboratory results for OGTT, glycosylated hemoglobin, and lipids. If a two-step approach to screening is chosen, it serves both parts. Importantly, in the final stage, the laboratory result is used as a continuous variable in combination with clinical findings indicating greater or lesser risk.

One diagnostic property not described in the comparison of the three screening approaches was the probability of missing high-risk individuals using the BrDMrisc. However, given that most individuals are not deemed high risk by all strategies, this probability is always low (Figure 1). While the likelihood that an individual not labeled high-risk would develop diabetes in the ensuing 10 years was not zero, their (post-test) probability of developing diabetes was less than 10%. Further, repeat screening over intervals of a few years mitigates the risk of missing future cases in the population, making current identification of lower-risk individuals less necessary.

Despite these strengths of the ELSA-Brasil study and the calculator, a limitation meriting mention is the incomplete investigation of the score’s generalizability. Despite the presence, in our cohort, of large numbers of participants across categories of the principal domains of Brazilian society—age, sex, ethnicity, schooling level, and income—and our internal validation of the prediction scores, external validation in other samples would further strengthen support for their capacity for use in other settings.

In conclusion, the BrDMrisc approach to screening appears to be superior to other available strategies and readily available for use in Brazil and other parts of the developing world where diabetes is an increasing health problem.

## Data Availability

Following protocols established for ELSA-Brasil based on recommendations made by the ethics committees of each of the study’s institutions, the deidentified data used in this study can be made available for research proposals by request to the ELSA Datacenter (estatisticaelsa@gmail.com) and the ELSA Publications Committee. The study protocol is public. The informed consent statement and statistical analysis plan are available upon request to the corresponding author.

## Footnotes

a Available from: https://elsabrasil.org/funcoes-de-risco/risco-diabetes-10-anos/
